# Preparation and Performance Analysis of Ceramsite Asphalt Mixture with Phase-Change Material

**DOI:** 10.3390/ma15176021

**Published:** 2022-08-31

**Authors:** Jun Yuan, Peidong He, Haiyang Li, Xuesong Xu, Weiwei Sun

**Affiliations:** 1 School of Civil Engineering, Nanjing Forestry University, Nanjing 210037, China; 2 Department of Civil Engineering, Nanjing University of Science and Technology, Nanjing 210094, China

**Keywords:** phase-change materials, ceramsite, asphalt mixture, road performance, heat absorption, heat resistance

## Abstract

In this paper, phase-change material (PCM) and ceramsite were used to increase the heat resistance of the asphalt mixture. The ceramsite asphalt mixture with PCM can bring a specific cooling effect to the road surface and alleviate the rapid deterioration at high temperature. Two phase-change materials, PCM-43 and PCM-48, were compared and selected as the heat absorption material of the asphalt mixture. It is found that PCM-43 has better thermal stability, temperature regulation performance, higher enthalpy value, and a less adverse effect on the rheological properties of asphalt. According to the road performance of the asphalt mixture, it suggests that the maximum content of ceramsite is 40%. The specific heat capacity of asphalt mixtures was studied by the method of the insulation bucket test, and the thermal conductivity coefficient of asphalt mixtures was tested by a thermal conductivity instrument. The results show that the specific heat capacity and thermal conductivity of the asphalt mixture can be reduced by adding PCM and ceramsite. The effect of ceramsite asphalt concrete with PCM on the temperature field of road structure was further analyzed by finite element method. Due to the thermal resistance of ceramsite in the upper layer, the cooling range and depth in the middle and lower surface layers can be improved. Meanwhile, the heat absorption of phase-change material can alleviate the heating phenomenon of the upper layer. Therefore, ceramsite asphalt concrete with PCM is effective for decreasing the high temperatures in the asphalt pavements.

## 1. Introduction

Asphalt concrete pavements represent an essential role of construction materials in urban areas. However, in summer, the surface temperature of asphalt concrete pavement increases quickly due to high solar radiation and thermal convection between the pavement surface and the air. As a result, rutting will further accelerate pavement damage [1,2]. Meanwhile, it will aggravate urban heat island effect [3,4].

Nowadays, some cooling methods have been proposed to reduce pavement temperature [5,6,7]. One method is to improve the high-temperature stability of asphalt and asphalt mixture, such as using modified asphalt or adjusting the gradation of the asphalt mixture. Another method is to utilize a variety of cooling road surfaces, such as thermal resistance road surface and thermal reflection road surface, to achieve the cooling effect. Heat reflective coating with high reflectivity materials can reduce the absorption of solar radiation [8]. Thermal resistance aggregates with a low conductivity, such as waste ceramic tile [9] and shale ceramsite [10], can reduce the thermal conductivity of asphalt mixture and decrease the temperature of the pavement. By using ceramsite, the maximum surface temperature drop range of the indoor asphalt pavement specimen test could reach 7 °C [10]. The thermal resistance aggregate has some advantages for its porous structure and low thermal conductivity. Furthermore, using ceramsite can protect the environment and reduce the damage to the mountain due to stone quarrying. Nevertheless, at the same time, ceramsite has a low compressive strength and a large asphalt absorption ratio. These characteristics will limit its amount in the asphalt mixture.

The methods mentioned above are passive cooling. In recent years, some researchers also opted for the active cooling method to reduce the pavement temperature, such as adding phase-change material (PCM) to the asphalt mixture [11,12]. PCM is a material that absorbs or releases heat through the phase transition in the range of phase-change temperature [13,14]. After adding PCM to asphalt concrete, the PCM keeps its temperature stable when the environment temperature reaches the phase-change temperature and absorbs ambient heat until the end of phase-change heat absorption [15]. When PCM absorbs heat, it can be regarded as increasing the specific heat capacity of PCM itself [16]. In this way, the specific heat capacity of the asphalt mixture with PCM increases in the phase-change temperature range of PCM, which can be increased by 43% at 278.15 k [17]. It can effectively reduce the heating rate of pavement and alleviate high-temperature distresses.

In this study, active and passive cooling methods are simultaneously considered. So PCM and ceramsite were mixed into asphalt concrete to prepare a thermal resistance asphalt mixture. It aims at investigating the thermal storage properties of PCM and ceramsite on the latent heat storage capacity of asphalt mixture. The feasibility of PCM in asphalt was proved through various experiments, included FTIR test, DSC test, TG test for PCM, DSR test for asphalt, and the Marshall test for the mixture. Then, road performance and heat resistance performance of thermal resistance asphalt mixtures were studied. What is more, the temperature field of the pavement structure affected by ceramsite asphalt mixture with PCM is analyzed by the finite element method.

## 2. Materials and Methods

### 2.1. Raw Materials

**Figure 1:**
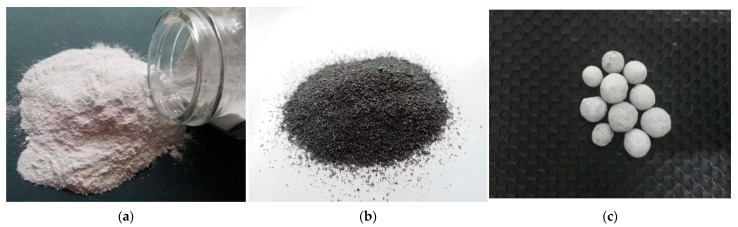
Raw materials: (**a**) PCM-43; (**b**) PCM-48; (**c**) ceramsite.

The study used a base asphalt binder, PG64-22, high-quality basalt aggregates, and limestone as mineral filler. All technical indices of the above materials achieved the requirements of the Technical Specification for Construction of Highway Asphalt Pavement. AC-13 asphalt mixture was selected in this study, and the aggregate gradation is shown in Table 1. The phase-change materials were obtained from Shanghai Xinya New Material Technology Co, Ltd. and named PCM-43 (Figure 1a) and PCM-48 (Figure 1b) according to their phase-change temperature. The ceramsite with particle sizes between 4.75 mm and 9.5 mm was obtained from the Anhui Province of China (Figure 1c). The compressive strength of the ceramsite was 12.4 MPa, and its density was 1.84 g/cm^3^.

### 2.2. Preparation of Materials

The high-speed shearing method was utilized to prepare PCM-modified asphalt. Three different contents (1%, 5%, and 9% by weight of asphalt binder) of two PCMs were added to the asphalt at the temperature of 160 °C. Then, the composites were stirred by continuous shearing for 30 min at a speed of 1200 rpm.

PCM and ceramsite were used to prepare the AC-13 asphalt mixture. According to Table 2, the optimum proportion of asphalt was 4.8%. The optimized mixing amount of phase-change material was 1% of the mix mass. The ceramsite particles substitute the equal volume of coarse aggregates at 0%, 20%, 40%, and 60%, respectively.

### 2.3. Test Methods

#### 2.3.1. Thermal Reliability Performance Evaluation

A VERTEX 80 V infrared spectrometer from Brooke, Germany, was used to analyze whether new chemical bonds and functional groups of PCM generate after high-temperature heating. The wave number range of the test was controlled at 500 cm^−1^~4000 cm^−1^.

A thermogravimetric analyzer (TG209F1, NETZSCH, Selb, Germany) and differential scanning calorimeter (DSC214, NETZSCH, Selb, Germany) were used to gain the phase-change temperature range, enthalpy, and mass loss rate at 200 °C of PCM. The DSC test was preceded by heating the PCM to 150 °C for the elimination of the thermal history. After that, it was cooled down from 150 °C to 15 °C with nitrogen. Then, the DSC test was carried out. The TG test samples weighed approximately 10 mg. By raising the testing temperature from room temperature (20 °C) to 200 °C at a rate of 10 °C/min in the nitrogen atmosphere, the mass transformation of the PCMs was recorded.

#### 2.3.2. Rheological Properties of Asphalt with PCM

In this study, the DSR (MCR-102, Anton Paar, Ostfildern, Germany) was used to evaluate the rheological properties of asphalt with PCM. The temperature of the tests was from 46 °C to 82 °C, and the load was ω = 10 rad/s. Three indicators, the complex shear modulus (G*), phase angle (δ), and rutting factor (|G*|/sinδ), of asphalt with PCM were measured at 6 °C intervals. The dosage of the PCM were 0%, 1%, 5%, and 9%, respectively.

#### 2.3.3. Temperature Regulation Performance of PCM

**Figure 2:**
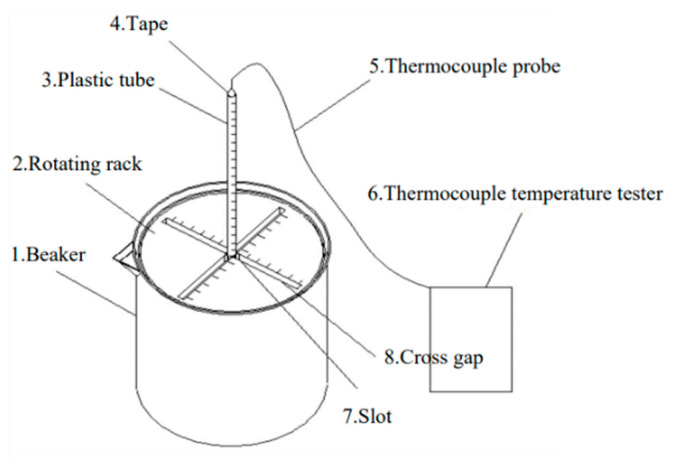
Schematic diagram of the temperature measurement device.

Since the existing devices were not very convenient in measuring the temperature regulation performance of asphalt with PCM, a device was invented (Figure 2). Firstly, the PCM asphalt was heated to the flow state and put into the beaker. A rotating frame was placed above the beaker. The thermocouple detection line wrapped with a plastic tube was inserted into the PCM asphalt to measure the temperature. The thermocouple probe can be controlled in its plane position through the slot on the rotating frame and its depth in the PCM asphalt through the scale on the plastic tube. In this way, the center temperature of PCM asphalt can be measured by a thermocouple temperature tester.

#### 2.3.4. Performance of the Thermal Resistance Asphalt Mixture

The high temperature, low temperature, and water stability performance of PCM asphalt mixtures with different ceramsite contents (0%, 20%, 40%, 60%) were carried out. Then, the specific heat capacity of the thermal resistance asphalt mixture was tested by the method of the insulation bucket test, and the thermal conductivity of the asphalt mixture was measured by the thermal conductivity instrument.

### 2.4. Finite Element Analysis

In order to study the influence of phase-change material and ceramsite on the temperature field of asphalt concrete pavement, three structures are analyzed, as shown in Table 3. The main factors affecting the temperature field of asphalt pavement structure include solar radiation, air temperature, convective heat transfer, and the effective radiation of pavement. The temperature change of the road surface from 9:00 a.m. to 7:00 p.m. was simulated by finite element calculation. The initial steady-state temperature load value was set as 35 °C at 9:00 a.m. The constraint temperature of natural soil at the bottom of the model was 15 °C. The temperature field was calculated according to the measured thermophysical parameters of mixture (Table 4). 

## 3. Results and Discussion

### 3.1. Chemical Stability of PCM

**Figure 3:**
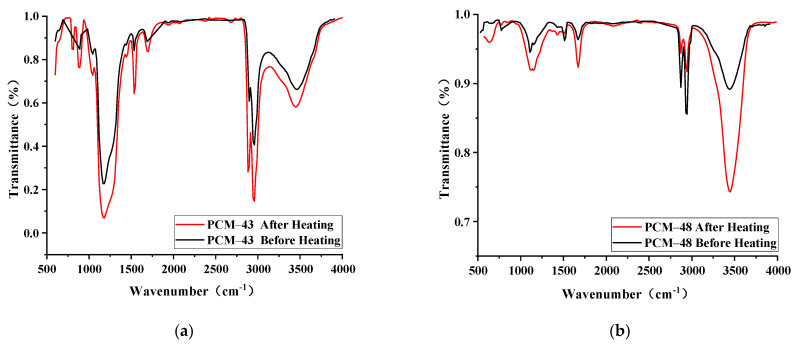
Comparison of transmittance change before and after heating: (**a**) PCM-43; (**b**) PCM-48.

In order to study whether the chemical composition of PCM-43 and PCM-48 would change after high-temperature heating, the infrared spectrum test was carried out on the phase-change materials before and after high-temperature heating (Figure 3).

The results indicate that the absorption peaks of the phase-change materials before and after high-temperature heating are basically in agreement. It proved that PCM-43 and –PCM-48 did not change their chemical composition and produce new functional groups after high-temperature heating. Thus, the PCM can be incorporated into the asphalt mixture successfully.

### 3.2. The Thermal Stability of PCM

**Figure 4:**
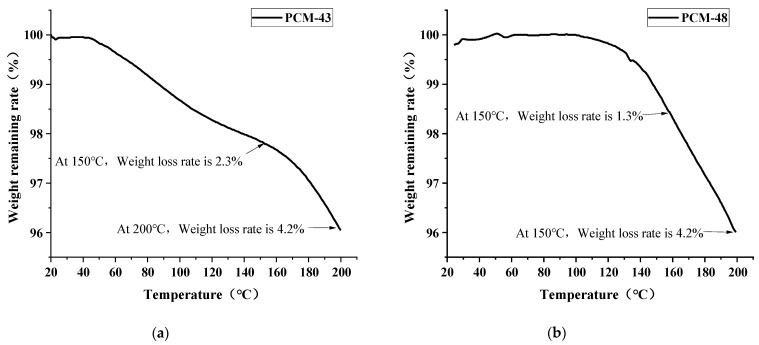
TG curve: (**a**) PCM-43; (**b**) PCM-48.

Thermal stability is an essential element in evaluating materials for temperature regulation applications in asphalt concrete. TG curves are presented in Figure 4 to evaluate the thermal durability property of PCM. As seen in Figure 4, PCM-43 is stable under 50 °C while PCM-48 has more excellent thermal stability under 130 °C. When the heating was continued, the mass loss started to occur; the loss rate of PCM-43 was 2.3% at 150 °C while PCM-48 was 1.3%. When the temperature reached 200 °C, PCM-43 nearly had the same mass loss rate as PCM-48. 

The results indicate that the two PCMs cannot easily volatilize within the phase-change temperature range. The mass loss of PCMs within 200 °C is minimal, while the mixing temperature of asphalt concrete is usually below 170 °C. It means the PCMs can remain stable within 200 °C and endure the high temperature of the mixing process. 

It can be found from the DSC test that during the heating process, the initial phase-change temperature and final phase-change temperature of PCM-43 were 36 °C and 50 °C, respectively, and the peak temperature of the phase change appeared at 43 °C; the exothermic enthalpy was 210 J/g. PCM-48 had similar properties. The starting point of the phase change was 42 °C, the ending point of the phase change was 55 °C, the peak temperature of the phase change was 48 °C, and the exothermic enthalpy value of the phase change was 120 J/g.

### 3.3. Effect of PCMs on the Rheological Properties of Asphalt

The dynamic shear rheology test was chosen to evaluate the effect of PCMs on the rheological properties of asphalt.

**Figure 5:**
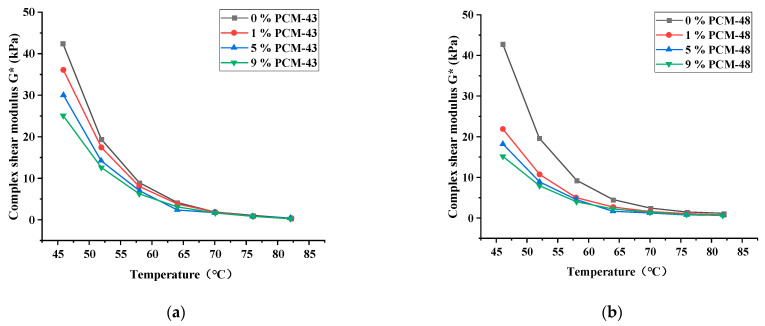
The complex modulus of asphalt with different dosages of PCM: (**a**) PCM-43; (**b**) PCM-48.

From Figure 5, it can be found that the complex shear modulus of the asphalt with different dosages of PCM decrease as the test temperature increases. It is due to the increasing free-flowing volume of the asphalt as the temperature rises, and the asphalt transforms from a highly elastic state to a viscous flow state. At the same temperature, the complex shear modulus of the asphalt decreases gradually with the increase of the dosage of PCM. This trend is consistent with the use of tetradecane as a PCM [18]. At the same PCM amount, the composite modulus of asphalt with PCM-43 reduces more significantly than with PCM-48.

**Figure 6:**
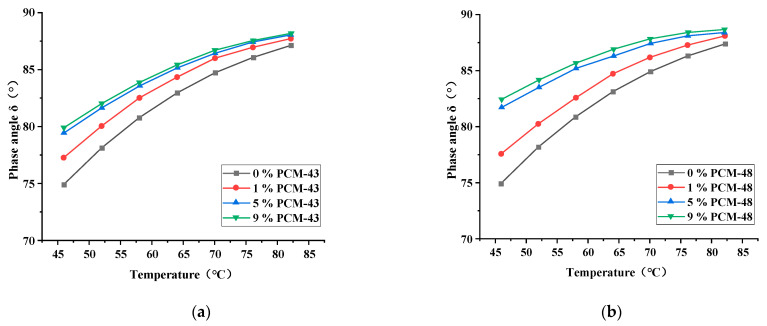
The phase angle of asphalt with different dosages of PCM: (**a**) PCM-43; (**b**) PCM-48.

With the increase of the temperature, the phase angles of the asphalt with different dosages of PCM gradually increase (Figure 6). This illustrates that the viscous part of the asphalt increases and the elastic part decreases after the temperature increases such that the phase angle of asphalt becomes larger [19]. The phase angles of two asphalts with PCM are larger than that of the base asphalt at any temperature. At the same dosage, the influence of PCM-48 on asphalt is slightly greater than PCM-43.

**Figure 7:**
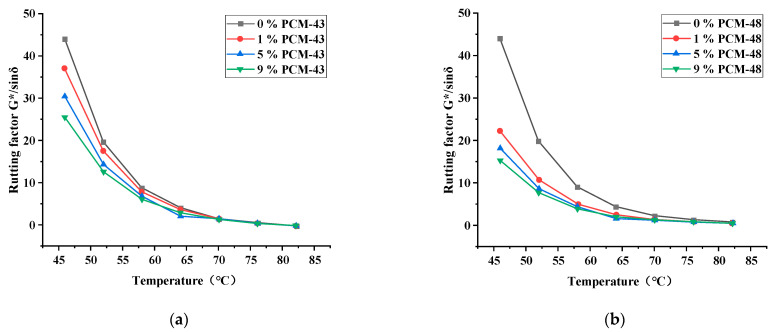
Rutting factor of asphalt with different dosages of PCM: (**a**) PCM-43; (**b**) PCM-48.

As seen in Figure 7, the trend of the rutting resistance factor is basically the same as the complex shear modulus. The test results indicate that the phase-change material has a slight adverse effect on the high-temperature performance of the asphalt. Comparing the two phase-change materials, PCM-43 has less influence on the high-temperature performance of asphalt. Additionally, the higher the amount of PCM, the lower the anti-rutting factor at high temperatures. This phenomenon is because the shell material of PCM impacts the high-temperature performance of asphalt, so the dosage of PCM should be controlled in the asphalt mixture.

### 3.4. Temperature Adjustment Effect of PCM

**Figure 8:**
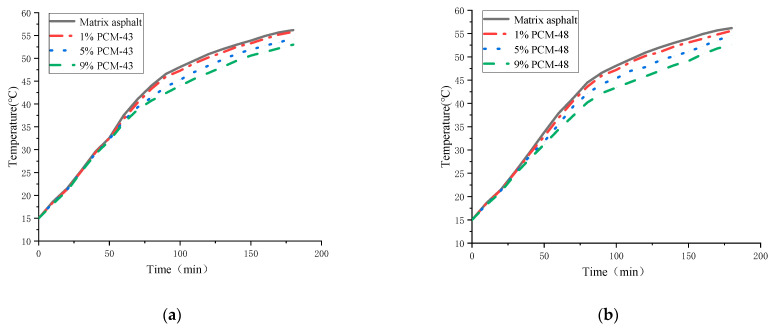
Temperature adjustment performance of PCM asphalt: (**a**) PCM-43; (**b**) PCM-48.

Figure 8 is the temperature adjustment effect diagram of asphalt mixed with PCM-43 and PCM-48.

It can be seen that two phase-change materials can both slow down the heating of asphalt to a certain extent in Figure 8. As the amount of phase-change materials increases, heating delaying is better. It can be found that the heating rate of all samples is almost the same in the initial heating stage. When approaching the phase transition point, the temperature difference gradually appears for the different dosages of PCM. Moreover, the temperature difference shows a trend of gradual expansion with the increase of PCM dosage.

The maximum temperature difference between the asphalt with 9% PCM-43 and base asphalt is 6 °C, while the maximum temperature difference between the asphalt with 9% PCM-48 and base asphalt is about 4 °C. In the previous DSC test, the enthalpy of PCM-43 was about 210 J/g, while the enthalpy of PCM-48 was only 120 J/g. The former is almost twice as high as the latter. It makes the temperature adjustment performance of asphalt with PCM-43 greater than that of asphalt with PCM-48 at the same dosage of phase-change material.

It can also be found that the asphalt with PCM-43 starts to produce a temperature difference at about 25 °C instead of at the phase-change point of 36 °C, and the asphalt with PCM-48 produces a temperature difference at about 34 °C instead of at its phase-change point of 42 °C. The reason for this phenomenon is that the specific heat capacity of the phase-change material is slightly higher than that of the asphalt, and when the phase-change material is incorporated into the asphalt, the specific heat capacity of the asphalt with PCM is larger than that of the base asphalt.

From the analysis of the results in Section 3.1, Section 3.2, Section 3.3 and Section 3.4, it is clear that PCM-43 has a better performance compared with PCM-48. Therefore, PCM-43 was added into the asphalt mixture to test its performance in the following test. PCM dosage is suggested as 1% of the asphalt mixture; this dosage was similar to that derived by Chen [20]. 

### 3.5. Road Performance of Ceramsite Asphalt Mixture Mixed with PCM

The road performance of PCM asphalt mixtures with different ceramsite content is shown in Table 4.

The technical requirements in Table 3 are based on the requirements of the upper surface layer in low-medium traffic volumes in Chinese national standard CJJ 169-2012. It can be seen from Table 2 that with the increase of ceramsite content, the dynamic stability of asphalt concrete declines. When the content of ceramsite achieves 60%, the dynamic stability can no longer meet the requirements of the specification. Although the ceramsite selected in this study are high-strength ceramic particles, the strength of ceramsite itself is still much lower than that of the basalt aggregate [21]. Therefore, the dynamic stability declines gradually with the increase of ceramsite dosage; that is, ceramsite negatively impacts the high-temperature stability of asphalt concrete.

With the increase of ceramsite content, the maximum bending tensile strain of the asphalt mixture decreases because the ceramsite becomes brittle after freezing [22,23], which weakens the low-temperature stability of asphalt concrete.

The water stability of the asphalt mixture is also in a declining trend with the increase of ceramsite content. The adhesion between ceramsite and asphalt is poor. Under the action of water, the asphalt film attached to the ceramsite surface will strip, which reduces the water stability [24]. In addition, due to the high water absorption rate and strong water retention of ceramsite, external water is easy to stay in the mixture. Finally, the mixture loosens under the action of a load [25]. When the content of ceramsite is 60%, the freezing-thawing splitting strength ratio of PCM asphalt mixture cannot meet the specification requirements.

To sum up, it is recommended that the dosage of ceramsite should not be over 40%.

### 3.6. Thermophysical Property of Ceramsite Asphalt Mixture with PCM

Since the specific heat capacity of PCM is larger than that of the basalt aggregate, the specific heat capacity of the asphalt mixture slightly increased after adding PCM to it. However, when ceramsite is used to replace the basalt aggregate in the asphalt mixture, with the increase of ceramsite content, the specific heat capacity of the asphalt mixture will gradually decrease (Table 5). It is due to the ceramsite having a lower specific heat capacity than the basalt aggregate. The thermal conductivity of PCM and ceramsite is lower than that of the basalt aggregate. When ceramsite is used to replace the basalt aggregate in the PCM asphalt mixture, the thermal conductivity of the asphalt mixture can be significantly reduced.

### 3.7. Temperature Conditions of Road Structures

**Figure 9:**
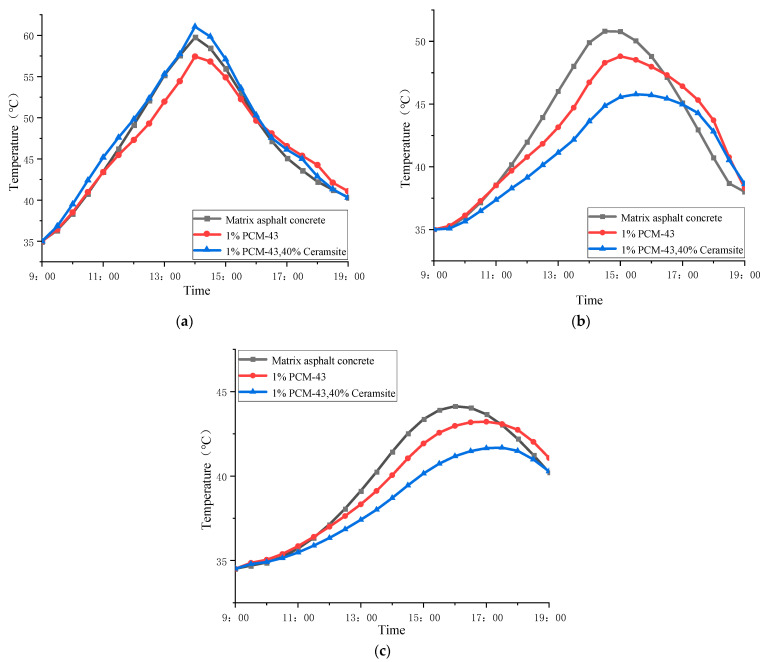
The temperature of pavement structure changing with time: (**a**) top of the upper layer; (**b**) top of the middle layer; (**c**) top of the lower layer.

After finite element calculations, the temperatures at the top of the upper, middle, and lower surface layers of the pavement structure were calculated for comparison, and the figure was shown in Figure 9.

It can be seen from Figure 9a that the maximum temperature at the top of the upper surface of the three road structures all occurs at about 2 p.m. The maximum temperature at the top of the upper surface of structure C reaches 61.05 °C, which is the largest compared with the other two structures. The maximum temperature at the top of the upper surface of structure B is 57.65 °C, which is the least. With the environment temperature rising, the phase-change material in the upper layer of structure B absorbs part of the heat, lowering the peak temperature of the road surface. On the top surface of the upper layer of structure C, due to the thermal resistance effect of ceramsite, the heat continuously accumulated in the road surface, thus increasing the road surface temperature. However, due to the endothermic effect of the phase-change material in the upper layer of structure C, the temperature rise rate of the road surface is buffered in the phase-change interval, so the maximum temperature of the road surface is only 1.3 °C higher than that of structure A; this increase is smaller than in the case of adding ceramsite alone [21]. With the decrease of the environment temperature, the surface cooling rate of structure A is the fastest. The phase-change materials in the upper layer of the other two road structures produce phase-change heat release in the phase-change interval, which prevents the rapid cooling of the road surface and avoids the sudden drop of the road’s temperature.

In Figure 9b, the maximum temperature at the top of the middle surface layer of structure A is the largest among the three structures, reaching 50.8 °C. The maximum temperature at the top of the middle surface layer of structure C is the lowest, 5.2 °C lower than that in structure A. In addition, the maximum temperature at the top of the middle surface layer of structure C occurs nearly one hour later than in structure A. This phenomenon is because of the dual effect of heat resistance of ceramsite and heat absorption of phase-change material in the upper layer of structure C. Thus, the temperature rising rate of structure C is slow. The temperature at the top of the middle surface layer in structure A still decreases most quickly, while structures B and C decrease slowly due to the effect of phase-change materials in the upper layer.

As shown in Figure 9c, the maximum temperature at the top of the lower surface of structure A is 44.13 °C, while that in structure C is 41.69 °C. The temperature difference between structure B and structure A is only 0.91 °C. It means that with the increase of the depth of the road structure, the heat absorption of the phase-change material and the heat resistance of ceramsite gradually begin to weaken.

**Figure 10:**
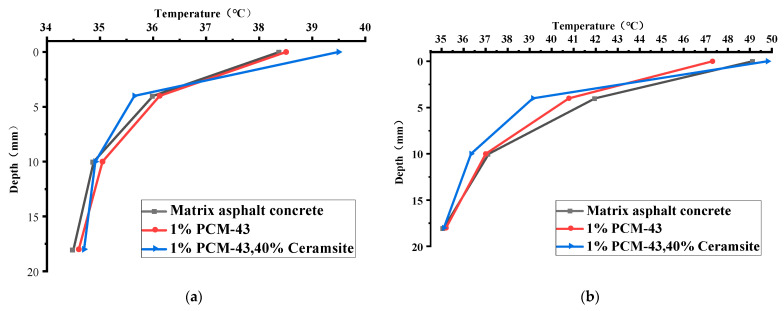

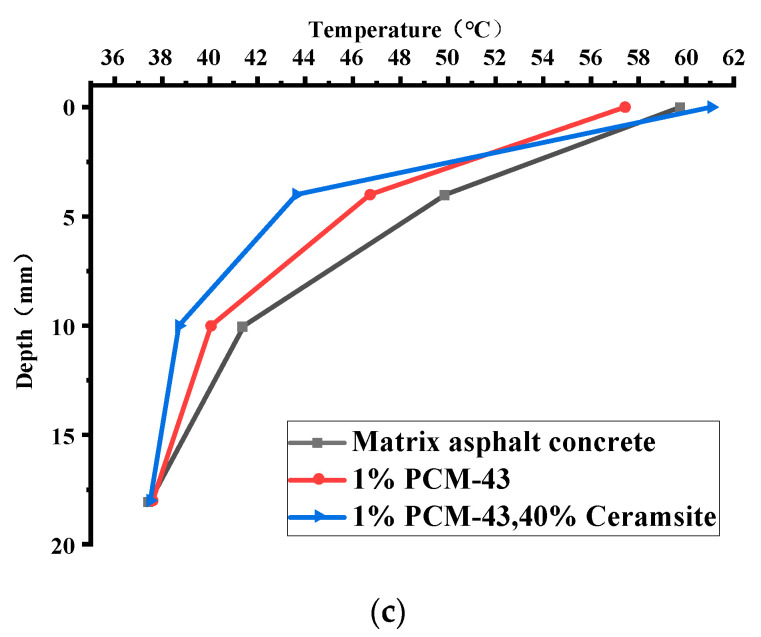
Temperature of pavement structure changing with depth: (**a**) 10:00; (**b**) 12:00; (**c**) 14:00.

The temperatures at different depths of the pavement structure layer at 10:00, 12:00, and 14:00 are plotted in Figure 10.

As can be seen from Figure 10, at 10 a.m., following the rise of environment temperature, ceramsite in the asphalt mixture produces a heat-resistance effect, which makes the upper surface layer temperature of structure C slightly higher than that of the other two road structures. Because the ceramsite blocks the heat source above the road and slows down the heat transfer, the temperature at the middle and lower surface layers of structure C is lower than that of the other two structures. The temperatures of the surface layers of structure A and structure B are close.

From 10 A.M. to 2 P.M., the environment temperature continues to rise and reaches the phase-change point of the phase-change material. The phase-change material gives full play and reduces the temperature of the road surface. It can be seen that the surface layer temperature of structure B is lower than that of structure A for the effect of the phase-change material at 12 P.M. In structure C, although the phase-change material has an endothermic effect, the temperature at a distance of 1–2 cm from the road surface is slightly higher due to the thermal resistance of ceramsite. However, the temperature at the rest of the surface layer is lower than that of the other two structures.

## 4. Conclusions

(1) Two PCM materials were compared, and the performance of PCM-43 is relatively superior, with higher enthalpy, lower mass loss rate at 200 °C, and a better temperature adjustment effect. However, with the increase of PCM content in asphalt, the rutting resistance factor decreases gradually, which is unfavorable to the high-temperature performance of asphalt.

(2) The strength of ceramsite is lower than that of the basalt aggregate, and ceramsite has large porosity and oil absorption, so the pavement performance of the asphalt mixture decreases when the basalt aggregate is replaced by ceramsite. Therefore, it is recommended that the content of the ceramsite should not exceed 40%. However, due to the addition of phase-change materials and ceramsite, the specific heat capacity and thermal conductivity of the asphalt mixture decrease gradually; that is, the thermal resistance of the mixture is improved.

(3) The temperature field of asphalt pavement with different kinds of asphalt mixture was simulated by the finite element method. When ceramsite asphalt mixture with PCM was used in the upper surface layer, it could realize the heat absorption ability of the phase-change material and heat-resistance ability of ceramsite at the same time. It will significantly delay the temperature rise of the asphalt pavement. The thermal resistance of ceramsite mitigated the further transfer of heat to the middle and lower layers of the road surface, while the heat absorption of the phase-change material lightened the heat accumulation in the upper layer of the road surface. Using ceramsite asphalt concrete with PCM can alleviate the high-temperature pavement distresses and slow down the urban heat island effect.

## 5. Patents

A test device for measuring the temperature of asphalt doped with phase-change materials, Utility Model, Granted, 2021, China Patent No. ZL2020212064039.

## Figures and Tables

**Table 1:** Aggregate gradation.

**SIZE (mm)**	16	13.2	9.5	4.75	2.36	1.18	0.6	0.3	0.15	0.075
**Passing rate (%)**	100	96	80	47	32.5	21	15	11	7	5

**Table 2:** Marshall characteristics of asphalt mixture.

Asphalt Content Variation (%)	Density	Relative Density	VV	VMA	VFA	Stability	Flow Value
	(cm^3^)	(cm^3^)	(%)	(%)	(%)	(KN)	(mm)
4.0	2.697	2.517	6.67	15.1	44.2	10.31	3.23
4.5	2.682	2.528	5.76	15.3	62.4	11.12	3.58
5.0	2.672	2.535	5.14	15.5	66.8	12.05	3.74
5.5	2.643	2.524	4.52	16.0	71.7	11.04	4.13
6.0	2.619	2.516	4.04	16.3	75.2	10.15	4.34

**Table 3:** Schematic diagram of the road structure.

Structure A	Structure B	Structure C
4 cm AC-13	4 cm AC-13 with PCM and 0% ceramsite	4 cm AC-13 with PCM and 40% ceramsite
6 cm AC-16	6 cm AC-16	6 cm AC-16
8 cm AC-25	8 cm AC-25	8 cm AC-25
40 cm Cement-stabilized base	40 cm Cement-stabilized base	40 cm Cement-stabilized base
3 m Foundation base	3 m Foundation base	3 m Foundation base
Natural Soil	Natural Soil	Natural Soil

**Table 4:** Road performance of PCM asphalt mixtures.

Ceramsite Content (%)	Dynamic Stability	$\mathrm{Maximum}\;\mathrm{Bending}\;\mathrm{Tensile}\;\mathrm{Strain}\;\varepsilon_{B}\left(u\varepsilon \right)$	Water Immersion Residual Stability MS_0_ (%)	Freeze-Thaw Splitting Strength Ratio (%)
0	1044	2523	88.1	84.38
20	911	2408	85.8	82.31
40	849	2236	86.5	78.24
60	778	2116	82.4	74.72
Technical requirements	≥800		≥80	≥75

**Table 5:** Thermophysical property of different kinds of asphalt mixture.

Mixture Type	Base Asphalt Concrete	PCM Asphalt Concrete with Ceramsite		
		0%	20%	40%
Specific heat capacity/ J/(kg·°C)	914	924	884	860
Thermal conductivity/ W/(m-°C)	1.27	1.21	1.01	0.81

## Data Availability

Not applicable.

## References

[1] Luo W., Li B., Zhang Y., Yin B., Dai J. (2020). A Creep Model of Asphalt Mixture Based on Variable Order Fractional Derivative. Appl. Sci..

[2] Mwanza A., Muya M., Hao P. (2016). Towards modeling rutting for asphalt pavements in hot climates. J. Civ. Eng. Archit..

[3] Cheela V., John M., Biswas W., Sarker P. (2021). Combating Urban Heat Island Effect—A Review of Reflective Pavements and Tree Shading Strategies. Buildings.

[4] Xu X., Swei O., Xu L., Schlosser C.A., Gregory J., Kirchain R. (2020). Quantifying Location-Specific Impacts of Pavement Albedo on Radiative Forcing Using an Analytical Approach. Environ. Sci. Technol..

[5] Anupam B.R., Sahoo U.C., Chandrappa A.K., Rath P. (2021). Emerging technologies in cool pavements: A review. Constr. Build. Mater..

[6] Jiang Y., Deng C., Chen Z., Tian Y. (2018). Evaluation of the cooling effect and anti-rutting performance of thermally resistant and heat-reflective pavement. Int. J. Pavement Eng..

[7] Zheng N.X., Lei J.A., Wang S.B., Li Z.F., Chen X.B. (2020). Influence of Heat Reflective Coating on the Cooling and Pavement Per-formance of Large Void Asphalt Pavement. Coatings.

[8] Zhang Y., Long E., Li Y., Li P. (2017). Solar radiation reflective coating material on building envelopes: Heat transfer analysis and cooling energy saving. Energy Explor. Exploit..

[9] Silvestre R., Medel E., Garcia A., Navas J. (2013). Using ceramic wastes from tile industry as a partial substitute of natural aggregates in hot mix asphalt binder courses. Constr. Build. Mater..

[10] Wang C., Fu H., Fan Z., Li T. (2019). Utilization and properties of road thermal resistance aggregates into asphalt mixture. Constr. Build. Mater..

[11] Guo M., Liang M., Jiao Y., Zhao W., Duan Y., Liu H. (2020). A review of phase change materials in asphalt binder and asphalt mixture. Constr. Build. Mater..

[12] Wang H.-R., Wang Y.-M., Qi M.-L., Wang Q.-L., Li X., Zhu X.-X., Yu X.-Q., Liu Z.-M. (2021). PEG/diatomite modified Asphalt Mixture with Self-Regulating temperature property. Case Stud. Constr. Mater..

[13] El Ouali A., El Rhafiki T., Kousksou T., Allouhi A., Mahdaoui M., Jamil A., Zeraouli Y. (2019). Heat transfer within mortar containing micro-encapsulated PCM: Numerical approach. Constr. Build. Mater..

[14] Mohseni E., Tang W., Wang S. (2019). Development of thermal energy storage lightweight structural cementitious composites by means of macro-encapsulated PCM. Constr. Build. Mater..

[15] Jiang J., Zhu Y., Ma A., Yang D., Lu F., Chen J., Shi J., Song D. (2012). Preparation and performances of bulk porous Al foams impregnated with phase-change-materials for thermal storage. Prog. Nat. Sci..

[16] Zhou D., Zhou Y., Yuan J., Liu Y. (2020). Palmitic Acid-Stearic Acid/Expanded Graphite as Form-Stable Composite Phase-Change Material for Latent Heat Thermal Energy Storage. J. Nanomater..

[17] Ren Y.-X., Hao P.-W. (2022). Low-Temperature Performance of Asphalt Mixtures Modified by Microencapsulated Phase Change Materials with Various Graphene Contents. Coatings.

[18] Kakar M.R., Refaa Z., Bueno M., Worlitschek J., Stamatiou A., Partl M.N. (2019). Investigating bitumen’s direct interaction with Tetradecane as potential phase change material for low temperature applications. Road Mater. Pavement Des..

[19] Wang C., Zhao L., Cao D. (2017). Experimental study on rheological characteristics and performance of high modulus asphalt binder with different modifiers. Constr. Build. Mater..

[20] Chen J., Zhang W., Shi X., Yao C., Kuai C. (2020). Use of PEG/SiO_2_ phase change composite to control porous asphalt concrete temperature. Constr. Build. Mater..

[21] Deng H., Deng D., Du Y., Lu X. (2019). Using Lightweight Materials to Enhance Thermal Resistance of Asphalt Mixture for Cooling Asphalt Pavement. Adv. Civ. Eng..

[22] Wang J., Zhang Z., Guo D., Xu C., Zhang K. (2018). Study on Cooling Effect and Pavement Performance of Thermal-Resistant Asphalt Mixture. Adv. Mater. Sci. Eng..

[23] Tian L., Qiu L., Li J., Yang Y. (2020). Experimental study of waste tire rubber, wood-plastic particles and shale ceramsite on the performance of self-compacting concrete. J. Renew. Mater..

[24] Muniandy R., Ismail D.H., Hassim S. (2018). Performance of recycled ceramic waste as aggregates in hot mix asphalt (HMA). J. Mater. Cycles Waste Manag..

[25] Javani M., Kashi E., Mohamadi S. (2019). Effect of polypropylene fibers and recycled glass on AC mixtures mechanical properties. Int. J. Pavement Res. Technol..

